# Evaluation of the relationship between oral glucose tolerance test, blood sugar levels and cholesterol levels in prediabetic individuals

**DOI:** 10.12669/pjms.40.5.8292

**Published:** 2024

**Authors:** Esra Aksoy Oduncu, Egemen Tural, Akin Dayan

**Affiliations:** 1 Esra Aksoy Oduncu, Family Medicine Clinic, Eyupsultan State Hospital, Istanbul, Turkey; 2 Egemen Tural, Department of Family Medicine, Haydarpasa, Numune Training and Research Hospital, Istanbul, Turkey; 3 Akin Dayan, Department of Family Medicine, Haydarpasa, Numune Training and Research Hospital, Istanbul, Turkey

**Keywords:** Oral glucose tolerance test, Triglyceride, HDL, Prediabetes, One-hour plasma glucose

## Abstract

**Objective:**

This study aimed to investigate whether there is a difference in lipid levels between prediabetic individuals with one-hour post-Oral Glucose Tolerance Test (OGTT) plasma glucose (PG) values > 155 mg/dl and those with one-hour PG values ≤ 155 mg/dl.

**Methods:**

This retrospective cross sectional study was initiated on August 2020 and concluded on June 2021, and conducted with 229 prediabetic patients who presented to the Diabetes Clinic of the Research Hospital. A correlation analysis was performed to investigate the relationship between OGTT values and serum lipid levels. Additionally, the patients were divided into two groups based on the one-hour post-OGTT PG value of 155 mg/dl, and the presence of any difference in serum lipid levels between the two groups was examined using the Mann-Whitney U test. The SPSS 20 software was used for statistical analysis, and a statistical significance level of P < 0.05 was considered.

**Results:**

Out of the 229 prediabetic patients included in the study, 172 were female. The number of patients with one-hour post-OGTT PG ≤ 155 mg/dl was 86, while those with values > 155 mg/dl were 143. A statistically significant difference was found between the group with one-hour post-OGTT PG > 155 mg/dl and the group with ≤ 155 mg/dl in terms of high-density lipoprotein (HDL-C) and triglyceride (TG) levels. There was a statistically significant negative correlation between one-hour PG and HDL-C.

**Conclusion:**

The evaluation of HDL-C and TG levels is important in prediabetic patients with a one-hour OGTT PG level greater than 155 mg/dL.

## INTRODUCTION

The identification of populations at high risk of developing diabetes is crucial for the prevention of diabetes. Prediabetic individuals have a higher likelihood of developing diabetes in the future compared to the general population.^1^ In addition, it has been shown that prediabetic individuals are more prone to cardiovascular diseases compared to individuals with normal glycemic control in different populations.^2^ The available evidence indicates that modifications in lifestyle and the implementation of pharmacological interventions have the potential to prevent and delay the progression of prediabetes to diabetes.^3^

Research has indicated that elevated blood cholesterol levels can lead to dysfunction of pancreatic beta cells.^4^ Beta cell dysfunction plays a significant role in the development of Type 2 diabetes mellitus (T2DM).^5^ Prediabetic individuals often have high plasma cholesterol levels, and controlling plasma cholesterol levels can reduce the risk of developing diabetes considering the adverse effect of hyperlipidemia on glycemic regulation. Therefore, screening and correction of lipid disorders in the early stages are recommended for the prevention of T2DM.^6^

In previous studies, it has been shown that a one-hour post-OGTT PG > 155 mg/dl is a better indicator for the progression from prediabetes to diabetes than the standard prediabetes criteria.^7^,^8^ Furthermore, the one-hour post-OGTT PG value has been found to be associated with cardiovascular risk and mortality.^9^

The aim of this study was to determine whether there are differences in lipid levels (total cholesterol, triglyceride, HDL-C, LDL-C) between prediabetic individuals with a one-hour post-OGTT PG value >155 mg/dl and those with ≤155 mg/dl. Additionally, the relationship between the OGTT fasting, one-hour, and two-hour values and lipid levels (total cholesterol, triglyceride, HDL-C, LDL-C) in prediabetic individuals was investigated.

## METHODS

Our study was conducted as a retrospective cross-sectional study and included prediabetic patients aged 18 years and above who presented to the Diabetes Clinic of the Training and Research Hospital.

### Ethical Approval:

It was approved by the Medical Ethics Committee of the Traning and Research Hospital under the decision number 2021/72-3220 in March 2021, was initiated on August 2020 and concluded on June 2021.

Between August 2020 and June 2021, it was determined that 526 patients who applied to our clinic met the inclusion criteria. After conducting the sample size calculation, the minimum number of patients required for the study was calculated as 223. The study was conducted with 229 patients. Participants were included based on a review of their medical records. Fasting blood glucose, OGTT, and cholesterol tests were performed for all participants. Patients with fasting blood glucose levels between 100 mg/dl and 126 mg/dl and two-hour OGTT values below 200 mg/dl were included in the study. Patients with two-hour OGTT values above 200 mg/dl, those using medications that affect glucose and lipid metabolism (such as statins, steroids, metformin, etc.), patients with a history of alcohol dependency, thyroid dysfunction, liver and kidney insufficiency, those who had experienced cerebrovascular disease and myocardial infarction within the past six months, and patients with active cancer were excluded from the study.

The patients’ gender, height, weight, waist circumference, body mass index (BMI), systolic blood pressure (SBP), diastolic blood pressure (DBP), fasting blood glucose, OGTT one-hour and two-hour plasma glucose values, total cholesterol (TC), triglyceride (TG), HDL-C, LDL-C, TSH, creatinine levels, and smoking status were collected after obtaining necessary permissions and anonymized for data analysis. For the OGTT, venous blood samples were taken at 60 minutes and 120 minutes after orally administering 75 grams of glucose following a 12 hours fasting period. All biochemical tests were performed in an accredited laboratory. IBM SPSS Statistics 21.0 (IBM Corp. Released 2012. IBM SPSS Statistics for Windows, Version 21.0. Armonk, NY: IBM Corp.) Software was used for statistical analysis and calculations. The normal distribution of data was assessed using the Kolmogorov-Smirnov test, q-q plot, skewness, and kurtosis.

Categorical data were presented as frequencies and percentages, while continuous data were presented as median (minimum-maximum) values. Non-parametric Mann-Whitney U test was applied for pairwise comparisons of age, gender, smoking status, and laboratory tests between groups. Spearman correlation analysis was used to assess the relationships between parameters, and point-biserial correlation analysis was used to evaluate the relationship between OGTT values and gender. Logistic regression analysis was used to identify factors predicting one-hour glucose>155mg/dl levels. As independent variables, the model included age, gender, BMI, waist circumference, HDL-C, Triglycerides, LDL-C parameters. A significance level of p < 0.05 was considered statistically significant.

### Consent:

All procedures performed in the studies involving human participants were in accordance with the ethical standards of the institutional and/or national research committee and with the 1964 Helsinki Declaration and its later amendments or comparable ethical standards.

## RESULTS

A total of 229 patients who met the inclusion criteria were included in our study. Patients were divided into two groups based on their OGTT one-hour plasma glucose values: OGTT >155 mg/dl and OGTT ≤155 mg/dl. The OGTT >155 group consisted of 186 patients, while the OGTT ≤155 group consisted of 43 patients. There were no statistically significant differences between the groups in terms of gender and smoking status (p=0.290, p=0.777) (Table-I). The demographic and laboratory data analysis of the participants is presented in (Table-II). A weak positive correlation has been identified between gender and fasting blood sugar (r: 0.131, p: 0.047). There is a weak positive statistically significant relationship between gender, height, waist circumference, HDL, and one-hour plasma glucose (respectively, r: 0.155, p: 0.019; r: 0.135, p: 0.041). Additionally, a weak positive statistically significant relationship has been observed between age and two-hour plasma glucose (r: 0.148, p: 0.025). A weak negative correlation has been identified between height and one-hour plasma glucose, as well as HDL cholesterol and one-hour plasma glucose (respectively, r: -0.133, p: 0.044; r: -0.168, p: 0.011). Furthermore, a weak negative correlation has been observed between height and two-hour plasma glucose (r: -0.292, p < 0.001).The correlation analysis between OGTT values and demographic/laboratory variables is presented in Table-III.

**Table-I:** Comparison of gender and smoking status according to OGTT one-hour plasma glucose values.

	All Participants n (%)	OGTT One-hour Plasma Glucose>155mg/dl n (%)	OGTT One-hour Plasma Glucose≤155mg/dl n (%)	Test Statistics

				P*
***Gender***				
Female	172(75.1)	137 (73.7)	35 (81.4)	0,290
Male	57(24.9)	49 (26.3)	8 (18.6)	
***Smoking Status***				
Yes	59(25.8)	47(25.3)	23(27.9)	0,777
No	124(54.1)	100(53.7)	24(55.8)	
Quit	46(20.1)	39(21.0)	7(16.3)	

* The Chi-square test statistics.

**Table-II:** Demographic and laboratory data of the two groups divided by one-hour glucose value of 155 mg/dl.

	OGTT One-hour Plasma Glucose>155mg/dl n:186	OGTT One-hour Plasma Glucose ≤155mg/dl n:43	Test Statistics

	Median (Min-Max)	Median (Min-Max)	p*
Age (years)	50 (18-80)	49 (26-68)	0,338
Weight (kg)	80 (51-125)	79 (56-124)	0,861
Height(cm)	158,50 (142-187)	163 (146-180)	0,106
Waist circumference (cm)	100 (67-147)	97 (80-134)	0,193
BMI(kg/m^2^)	31,04(18,96-53,41)	30,96 (23,01-43,91)	0,473
SBP(mmHg)	133,50 (91-185)	134 (97-222)	0,594
DBP(mmHg)	80 (40-110)	76 (48-139)	0,313
Creatinine(mg/dl)	0,80 (0,46-1,30)	0,81 (0,54-1,19)	0,389
Total cholesterol (mg/dl)	213 (96-343)	214 (158-281)	0,924
Triglycerides (mg/dl)	142 (37-393)	117 (61-273)	0,016
HDL-C (mg/dl)	48 (24-90)	53 (29-93)	0,026
LDL-C (mg/dl)	129,50 (34-248)	130 (79-119)	0,960
TSH (mIu/L)	1,43 (0,43-4,43)	1,46 (0,42-4,11)	0,711
OGTT fasting plasma glucose(mg/dl)	108 (100-125)	105 (100-118)	0,001
OGTT one-hour plasma glucose(mg/dl)	205 (156-278)	130 (68-154)	<0.001
OGTT two-hour plasma glucose(mg/dl)	132 (45-198)	108 (60-153)	<0,001

* Mann-Whitney U Test Statistics.

**Table-III:** Correlation between OGTT values and other parameters.

	OGTT fasting plasma glucose		OGTT one-hour plasma glucose		OGTT two-hour plasma glucose	

	r	p	r	p	r	p
Age(years)	0,111	0,094	0,124	0,062	0,148*	0,025
^#^Gender	0,131*	0,047	0,155*	0,019	-0,080	0,229
Weight(kg)	0,121	0,067	0,030	0,654	-0,021	0,756
Height(cm)	0,006	0,926	-0,133*	0,044	-0,292**	<0,001
Waist circumference(cm)	0,094	0,155	0,135*	0,041	0,095	0,152
BMI(kg/m^2^)	0,082	0,216	0,098	0,139	0,127	0,055
SBP(mmHg)	0,092	0,165	0,090	0,174	0,009	0,888
DBP(mmHg)	-0,006	0,927	0,074	0,265	-0,110	0,097
Creatinine(mg/dl)	-0,004	0,953	0,044	0,503	-0,073	0,274
Total cholesterol(mg/dl)	0,003	0,960	-0,023	0,728	0,0001	0,998
Triglycerides(mg/dl)	-0,045	0,499	0,092	0,164	0,068	0,307
HDL-C(mg/dl)	-0,045	0,496	-0,168*	0,011	-0,039	0,560
LDL-C(mg/dl)	0,053	0,429	-0,015	0,825	-0,004	0,948
TSH(mIu/L)	0,027	0,680	-0,042	0,525	-0,013	0,843

#For the correlation analysis between gender and OGTT values, Point-Biserial Correlation was used. For the correlation analysis between other parameters and OGTT values, Spearman’s Rank-Order Correlation was used.

* p<0.05

** p<0.001.

The logistic regression analysis evaluated the factors predicting the one-hour glucose>155mg/dl in generated models. The Nagelkerke R^2^ values was 0.072. In predicting the factors influencing the changes in one -hour glucose, triglycerides found to be significant (Exp (B): 1.007, p: 0.048) (Table-IV).

**Table-IV:** Logistic regression analysis of factors predicting one-hour glucose >155 .

	B	S.E.	Exp(B)	p	95% CI.for Exp(B)
Constant	0.020	1.948	1.020	0.992	
Age	0.019	0.017	1.019	0.268	0.986-1.054
Gender (Female)	-0.256	0.443	0.774	0.563	0.325-1.844
BMI (kg/m^2^)	-0.014	0.056	0.986	0.807	0.885-1.100
Waist circumference(cm)	0.011	0.026	1.012	0.655	0.962-1.064
HDL-C(mg/dl)	-0.009	0.014	0.991	0.526	0.965-1.018
LDL-C(mg/dl)	-0.004	0.005	0.996	0.410	0.986-1.006
Triglycerides(mg/dl)	0.007	0.004	1.007	0.048*	1.000-1.015

Cox & Snell R Square: 0.044, Nagelkerke R Square: 0.072.

* p<0.05.

## DISCUSSION

In our study, a significant difference was found between the group with OGTT one-hour PG >155 mg/dl and the group with ≤155 mg/dl in terms of HDL-C and TG. However, no significant differences were observed between the two groups in terms of LDL-C and total cholesterol (TC) levels. Additionally, a negative statistically significant correlation was found between one-hour PG and HDL-C. However, no significant correlation was found between fasting PG and two-hour PG levels with HDL-C.

In the study conducted by Farasat et al., it was mentioned that in the studied population, despite having normal total cholesterol levels, the low levels of HDL-C could indicate a lipid metabolism disorder, and this condition could potentially serve as a precursor to the development of T2DM.^10^ A study by Lizarzaburu-Robles et al. investigated the relationship between one-hour PG (>155 mg/dl) and metabolic syndrome in individuals with impaired fasting glucose (IFG). The study revealed that the risk of metabolic syndrome was twice as high in the group with one-hour PG >155 mg/dl compared to the group with ≤155 mg/dl.^11^ In our study, significant differences were found between the two groups divided by one-hour PG levels of 155 mg/dl in terms of fasting PG, TG, and HDL-C, which are components of the metabolic syndrome.

A study by Shimodaira et al. examined the relationship between one-hour PG levels and serum lipid levels. The study found a negative correlation between one-hour PG and HDL-C, and compared to individuals with one-hour PG levels ≤155 mg/dL, individuals with one-hour PG levels >155 mg/dL had higher TG levels and lower HDL-C levels. No significant differences were found between the two groups in terms of smoking habits.^12^ Our study is in line with Shimodaira’s findings.

In a study conducted by Akehi et al., involving 110 young male participants with normal glucose tolerance, the relationship between TG, TC levels, OGTT levels, and BMI groups (<22.18 kg/m², ≥22.18 kg/m²) was investigated. According to the results, both groups showed an association between one-hour and two-hour PG levels and TC levels. A correlation was found between TG and only one-hour PG, and this relationship was observed only in the group with higher BMI.^13^ In our study, no correlation was observed between TG and TC levels with one-hour PG. The difference in the results regarding the relationship between one-hour PG and TC/TG in our study compared to Akehi et al. may be attributed to the fact that their study focused only on male participants.

The study by Almari et al. focused on adolescent participants aged 14-19 years to examine the independent and combined effects of obesity and prediabetes on lipid levels.^14^ According to their results, obesity was associated with all investigated lipid levels independent of prediabetes. Decreased HDL-C and increased LDL-C were found to be associated with prediabetes independent of obesity. In our study, we only found a negative significant correlation between one-hour PG and HDL-C. No correlation was found between fasting PG and two-hour PG with HDL-C and LDL-C. The differences in the populations included in the studies and the variety of factors affecting lipid and glucose metabolism may explain the discrepancy between our study and Almari et al.’s findings.

In our study, a negative correlation was found between one-hour and two-hour PG levels and height. Negative associations between OGTT two-hour PG levels and height have been demonstrated in various populations. In a population-based study conducted by Pan et al., investigating the relationship between OGTT and height in individuals with impaired fasting glucose, similar to our findings, a negative correlation was found between OGTT two-hour PG levels and height.^15^

In another study conducted by Bhowmik et al, it was observed that prediabetic and T2DM participants were older compared to the normoglycemic group? Additionally, increased waist circumference and increased BMI were found to be associated with prediabetes and T2DM.^6^ In our study, it was observed that age was correlated with two-hour PG, and waist circumference was correlated with one-hour PG. The differences between groups in this study support our findings.

Prediabetes is a risk factor for cardiovascular diseases.^2^ A meta-analysis of 53 prospective cohort studies demonstrated a significant association between impaired fasting glucose (IFG) and impaired glucose tolerance (IGT) with cardiovascular mortality.^16^ Dyslipidemia, especially low HDL-C and high triglycerides, in individuals with prediabetes increases the risk of cardiovascular mortality.^17^,^18^ In a study conducted in Finland, a significant association was found between triglyceride levels and cardiovascular death risk in individuals with IFG and IGT. The significant association between one-hour PG and low HDL-C observed in our study suggests that one-hour PG levels may be important for assessing cardiovascular disease risk in prediabetic individuals. Furthermore, the group with one-hour PG >155 mg/dl having lower HDL-C and higher TG values compared to the group with lower PG supports this assumption.

### Limitations of the Study:

The primary limitation of our study is its cross-sectional design, which restricts our ability to establish causal relationships between variables. As a result, we cannot determine the cause-and-effect relationships between elevated one-hour post-load plasma glucose levels (>155 mg/dl) and the development of diabetes or the risk of cardiovascular disease. Additionally, insulin resistance has not been evaluated in our study because patients’ insulin values were not accessible.

## CONCLUSION

The results demonstrated a negative correlation between one-hour post-OGTT plasma PG values and HDL cholesterol levels. In our study, the absence of a relationship between fasting PG and two-hour PG levels with HDL-C and triglyceride levels, and the presence of the relationship with one-hour PG levels and HDL-C and triglyceride levels, can be considered as a significant factor emphasizing the importance of the subject. Additionally, lower HDL cholesterol and higher triglyceride levels were observed in the prediabetic group with one-hour PG values >155 mg/dl, which supports the existing literature indicating the significance of one-hour PG values in cardiovascular risk.

Therefore, careful monitoring of patients with high one-hour PG values, accompanied by low HDL-C and high triglyceride levels, is recommended in terms of cardiovascular risk. Furthermore, assessing one-hour PG values in the follow-up of patients with low HDL and high triglycerides may be important for detecting impaired glucose regulation.

### Author Contributions:

**EAO:** Concept and design, edited and reviewed the manuscript. Responsible and accountable for the accuracy and integrity of the work.

**ET:** Researched data, wrote and reviewed the manuscript.

**AD:** Designed, edited and reviewed the manuscript.
